# Influence of Post Weld Heat Treatment on the Grain Size, and Mechanical Properties of the Alloy-800H Rotary Friction Weld Joints

**DOI:** 10.3390/ma14164366

**Published:** 2021-08-04

**Authors:** Saqib Anwar, Ateekh Ur Rehman, Yusuf Usmani, Ali M. Al-Samhan

**Affiliations:** Department of Industrial Engineering, College of Engineering, King Saud University, Riyadh 11451, Saudi Arabia; sanwar@ksu.edu.sa (S.A.); yusmani@ksu.edu.sa (Y.U.); asamhan@ksu.edu.sa (A.M.A.-S.)

**Keywords:** friction welding, nickel alloys, alloy 800H, minimum grain size, heat treatment

## Abstract

This study evaluated the microstructure, grain size, and mechanical properties of the alloy 800H rotary friction welds in as-welded and post-weld heat-treated conditions. The standards for the alloy 800H not only specify the composition and mechanical properties but also the minimum grain sizes. This is because these alloys are mostly used in creep resisting applications. The dynamic recrystallization of the highly strained and plasticized material during friction welding resulted in the fine grain structure (20 ± 2 µm) in the weld zone. However, a small increase in grain size was observed in the heat-affected zone of the weldment with a slight decrease in hardness compared to the base metal. Post-weld solution heat treatment (PWHT) of the friction weld joints increased the grain size (42 ± 4 µm) in the weld zone. Both as-welded and post-weld solution heat-treated friction weld joints failed in the heat-affected zone during the room temperature tensile testing and showed a lower yield strength and ultimate tensile strength than the base metal. A fracture analysis of the failed tensile samples revealed ductile fracture features. However, in high-temperature tensile testing, post-weld solution heat-treated joints exhibited superior elongation and strength compared to the as-welded joints due to the increase in the grain size of the weld metal. It was demonstrated in this study that the minimum grain size requirement of the alloy 800H friction weld joints could be successfully met by PWHT with improved strength and elongation, especially at high temperatures.

## 1. Introduction

Nickel-based superalloys are ideal materials for engineering structures that require high strength, creep, corrosion, and oxidation resistance at ambient and elevated temperatures [1,2]. Popular high strength Ni-based alloys for high-temperature applications derive their strength from the fine precipitation of the Ni3X phase (X = Al, Ti, and Nb) in the FCC-austenitic matrix [3]. Corrosion and oxidation resistance are generally achieved by the addition of Cr as an alloying element to the Ni-based super alloys. On the contrary, solid solution strengthened Ni-based alloys with a combination of very high corrosion resistance and moderate strength find a wide range of applications in power generation, chemical processing, the petrochemical industry, and other specialty industries [4]. Relatively less expensive iron-based Fe-Ni-Cr alloys with nickel content in the range of 30–45% show superior corrosion resistance compared to the stainless steels [4]. The alloy 800, an iron-based Fe-Ni-Cr alloy, has replaced the expensive and high Ni-containing alloy 600 during the early 1960s for long-term creep applications at moderate operating stress conditions. The alloy 800H is a modified grade of the alloy 800, which restricts the carbon content in the range of 0.05–0.1 wt.%. Though the alloy 800H contains carbides (Ti and Cr) and nitrides (Ti) in the microstructure, due to a small carbon content specification, the alloy 800H is mainly strengthened by solid solution strengthening. Lately, the alloy 800H is being considered for the development of several high-temperature components and large parts in the generation IV nuclear energy systems, replacing the alloys 617 and 230 [5].

Autogenous fusion welding and welding with the matching fillers of the alloy 800H are reported to suffer from hot cracking, either in the fusion zone or in the terminal weld crater [6]. Alloys that solidify directly as austenitic show a higher propensity towards hot cracking due to the lower solubility of the non-metallic impurities in the primary austenitic grains [7]. The segregation of non-metallic impurities depresses the melting point of the grain boundary liquid phase. It makes the weld metal susceptible to hot cracking due to the tensile stresses generated by solidification shrinkage [8]. Further, the formation of carbides due to the carbon enrichment at the grain boundaries in the alloy 800H autogenous full penetration welds is reported to be the reason for the cracking of weld metal during solidification. A thermal analysis of the weld metal revealed that the fusion welding of the alloy 800H with nonmatching fillers (e.g., the alloy 82) reduced the freezing range and improved the hot cracking resistance [8]. Several over-matching fillers for the fusion welding of the alloy 800H have been developed and commercially used to fabricate engineering structures [9]. However, the structural design often demands thin-section autogenous welding and welding with matching fillers to avoid galvanic corrosion and thermal fatigue. Matching fillers were developed for the alloy 800H by adding Nb to the base composition. The prolonged exposure of the alloy 800H fusion weldments to the γ’ precipitation temperatures in the as-welded condition, viz. 600–700 °C, showed the initiation of intergranular cracks in the heat affected zone (HAZ) and subsequent propagation along the fusion boundary [10]. However, a post-weld solution heat treatment (PWHT) of 1090 °C for 30 min followed by air cooling has markedly reduced the HAZ cracking sensitivity.

Solid-state welding techniques such as rotary friction and friction stir welding techniques produce the weld joints in the solid-state without melting the weld metal [11,12]. Heat is generated due to the friction between the surfaces that are in relative motion under compressive stress conditions [13]. A solid-state welding process eliminates the weldability issues arising from the solidification of the liquefied metal, as seen in the fusion welding techniques [14]. The nickel-based superalloy 718 was joined using a linear friction welding (LFW) technique, and it was reported that LFW yielded sound weld joints without the typical fusion welding defects such as voids, inclusions, and the liquation of low melting phases [15]. Anand et al. have developed solid-state 800H weld joints using a rotary friction welding technique using different welding parameters [16]. The dynamic recrystallization of the weld metal during the welding resulted in fine grains (<50 µm) in the weldment, which is lower than the specified minimum American society for testing and materials (ASTM) grain size number five (65 µm). Lower grain size values in the weldment make the structure unsuitable for long-term creep applications. In another study, the pin-to-tube friction weld joints of the alloy 800, a precursor grade to the alloy 800H, displayed a substantially lower (about 2% of the base metal life) creep rupture life. The fine grain structure in the weldment has resulted in the significantly shorter creep life of the weldments. However, a PWHT of 1107 °C for 15 min has been reported to restore the creep life, and the failure location was shifted to the base metal. Drabble et al. [17] also demonstrated that employing grain boundary engineering techniques on the alloy 800H plates (such as the variation of the deformation percentage, annealing temperature and time) resulted in higher resistance to the grain boundary diffusion. A limited number of reports have been published on the effects of solid-state welding on the microstructure and mechanical properties of the solid solution-strengthened austenitic alloy 800H. To the authors’ knowledge, there are no reports available on the effects of the post-weld heat treatment on the microstructure and mechanical properties of the alloy 800H friction welded joints. In this study, the alloy 800H cylindrical specimens were joined using a rotary friction welding technique, and the effects of a post-weld heat treatment cycle on the weld metal’s grain size and mechanical properties were investigated and reported. Optical and scanning electron microscopy techniques were employed for the microstructure, grain size, and fractured surface investigations. The Vickers microhardness and tensile testing techniques were employed for the mechanical performance evaluation.

## 2. Materials and Methods

Alloy 800H cylindrical specimens of 20 mm diameter and 100 mm length in mill-annealed condition were used in this study. The chemical composition of the alloy is listed in Table 1.

Before the friction welding, the rods of the alloy 800H were face machined perpendicular to the rotational axis and degreased with acetone. The welding was carried out using a continuous drive rotary friction welding machine (ETA Technology, Bangalore, India) with a maximum axial force of 150 kN. The welding procedure corresponding to the rotary friction welding machine has been explained in ref. [18]. A typical friction welding cycle comprises five process parameters: friction pressure, burn-off length or friction time, spindle speed, upset pressure, and upset time. The first three belong to the friction stage, and the last two belong to the upset stage of the weld cycle. In the friction stage, the necessary heat required for the bonding of the base metals is generated, whereas, in the upsetting stage, the bond is consolidated. Because of the restrictions on the availability of the materials and machine time, only a limited set of experiments were carried out. Based on the initial trials, the spindle speed, upset pressure, and upset time were frozen at one setting (see Table 2). Only the friction pressure, burn-off length, and upset pressure were varied in typical one-factor-at-a-time fashion. Friction pressures below 200 MPa were found to be insufficient, as they took a significantly long time in the friction stage. Furthermore, the flash appeared to be flaky, indicating that the heat generated was insufficient to make the base metals sufficiently plastically soft.

Further experiments were conducted, maintaining the friction pressure at 200 MPa and changing the burn-off length in 1 mm steps. At 4 mm, the flash appeared to have the best appearance. Beyond 4 mm, there was no perceptive advantage—in fact, the total loss in the base materials’ length seemed to be excessive and therefore unwarranted. Thus, the friction pressure and burn-off lengths were finalized at 200 MPa and 4 mm, respectively.

After the friction welding, the weld joints were post-weld solution heat-treated at 1000 °C for 15 min followed by furnace cooling. The friction welded samples were subjected to a standard metallography procedure to study the macrostructure using a Nikon SMZ745T stereomicroscope (Nikon Instruments Inc., New York, NY, USA) and to study the microstructure using an optical microscope (Leitz GMBH &CO. KG, Leitzstrasse 2, 73447 Oberkochen, Germany). The line intercept method was employed for the determination of the average grain sizes of the base metal, HAZ, and weld metal. A solution containing 4 g of copper sulfate and 20 mL of hydrochloric acid in 20 mL of distilled water was used to etch the metallographic samples, and the etching time was 30 s. The scanning electron microscope (SEM) VEGA 3LMV (TESCAN ORSAY HOLDING, Brno, Czech Republic) and an energy dispersive X-ray spectroscopy (EDS) chemical analyzer from Oxford instruments (Tubney Woods, Abingdon, UK) were employed for the microstructure and chemical analyses of the weld samples, respectively. X-ray diffraction (XRD) using PANalytical (Malvern, UK, X’pert powder XRD) was used to identify the phases present in the base and weld metals. The survey of the hardness across the weld joint interface at the weld center was carried out using a Vickers hardness machine (MMT-X Matsuzawa, Akita Prefecture, Kawabetoshima, Japan) with a diamond pyramid indenter under a load of 500 g for 15 s. ASTM E8 standard specimen configurations were employed for the tensile testing of the alloy 800H base metal, as-welded, and PWHT samples. Tensile tests were conducted using a servo-hydraulic testing machine (Instron, Norwood, MA, USA) at a constant displacement rate of 0.5 mm/min. Tensile testing was carried out at ambient and high temperatures (700 °C) to evaluate the weld joints’ mechanical performance.

## 3. Results and Discussion

### 3.1. Microstructure

**Figure 1:**
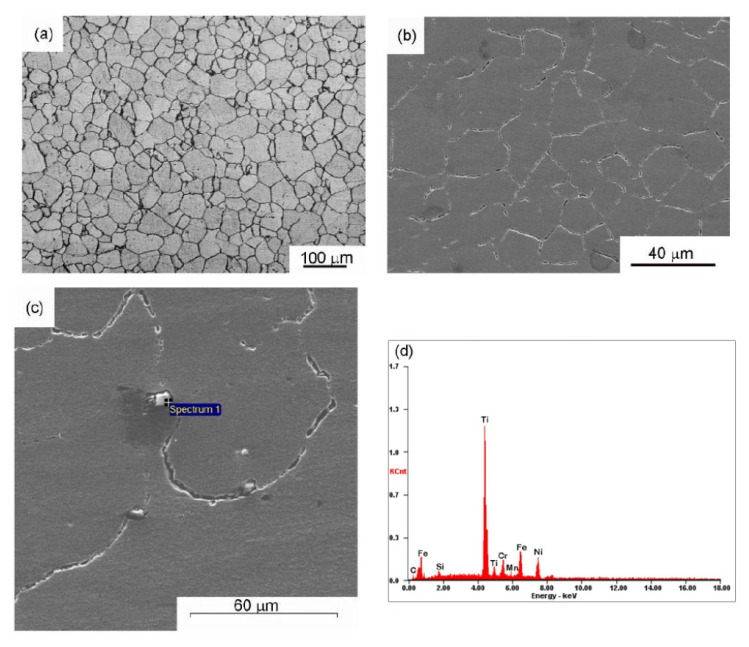
(**a**) Optical micrograph of the alloy 800H base metal, (**b**) secondary electron micrograph of the alloy 800H base metal, (**c**) SEM micrograph showing Ti-based precipitates, and (**d**) a typical EDS spectrum of precipitates.

The optical and scanning electron micrographs of the base metal alloy-800H are shown in Figure 1a,b. The micrographs showed equiaxed grains of the austenite phase. The average grain size of the base metal is 46 ± 7 µm. The higher magnification electron micrographs (Figure 1c) of the alloy 800H showed precipitates at the grain boundaries as well as within the grain interiors. A microchemical analysis under SEM revealed that the precipitates are rich in Ti and thus identified as Ti(C, N) precipitates (Figure 1d). 

**Figure 2:**
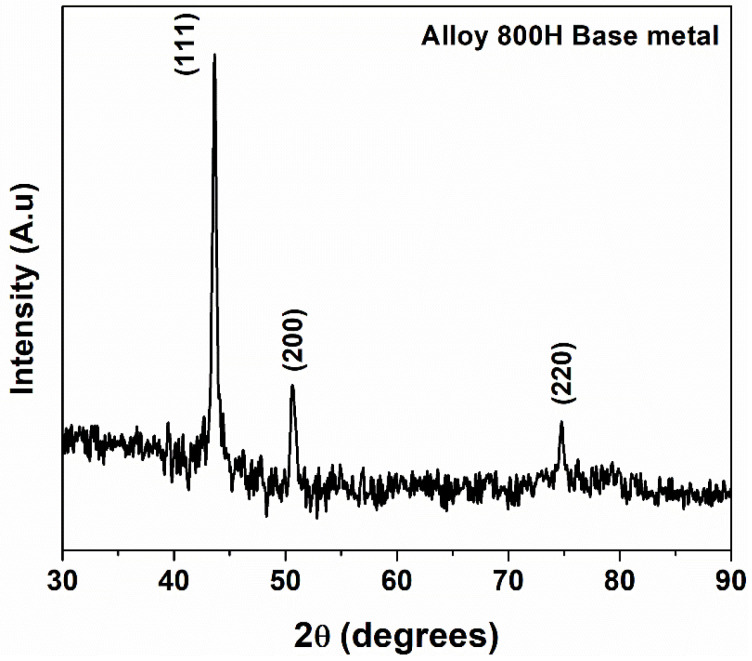
The XRD diffraction pattern of the alloy 800H base metal.

Similar Ti(C, N) precipitates were observed elsewhere in the solution annealed alloy 800H plates [6]. The XRD plot (Figure 2) of the base metal showed diffraction peaks corresponding to the single-phase austenite. Diffraction peaks from the secondary phases could not be seen due to the small volume fraction of the precipitates.

**Figure 3:**
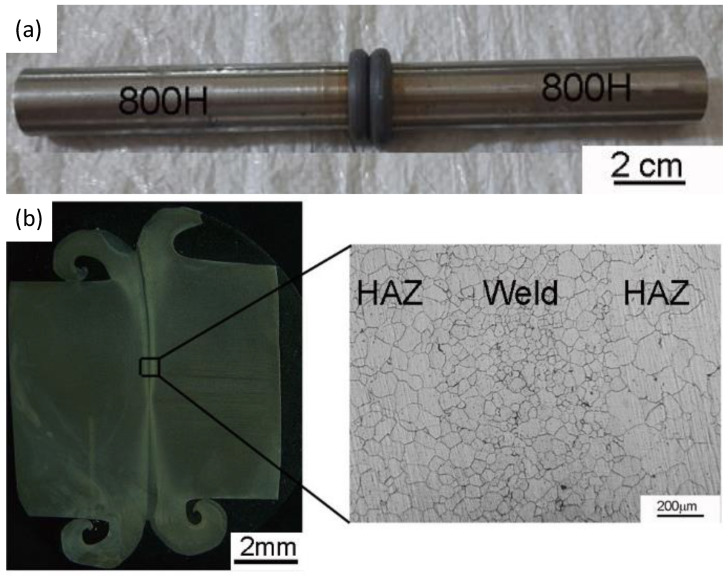
(**a**) The visual view of an Alloy 800H friction weld joint; (**b**) Macrograph of the friction weld joint with an inset showing the grain structure of the weldment.

The image of the typical friction welded joint of the alloy800H–alloy800H and the longitudinal weld cross sections’ macrograph are shown in Figure 3a,b, respectively. The welded joints showed a good flash, indicating that the sufficient plasticization of the materials at the joint area has taken place due to the heat generated by the frictional forces. The plasticized material contains native oxides and other contaminants from the base metal’s surface. The upset/forge pressure was sufficient to expel the plasticized material from the weld joint, thus producing the sound weld joint. A clean weld joint without delaminated areas or welding defects was obtained in this study, as evidenced by the longitudinal weld cross-sectional macrograph (Figure 3b).

**Figure 4:**
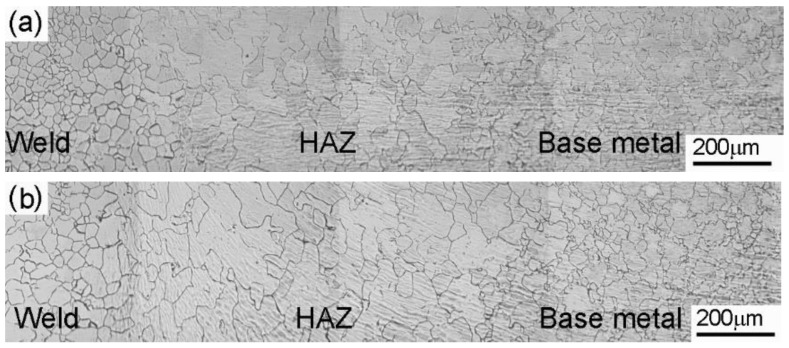
Optical micrograph of weldments; (**a**) as-welded condition; and (**b**) PWHT condition.

The optical micrograph of the weld cross-section (Figure 4a) shows three distinctive regions: the weld zone, the heat-affected zone (HAZ), and the base metal. The absence of deformed/elongated grains in the weld zone, as evidenced by the optical micrographs, indicates complete recrystallization of the deformed metal in the weld zone. Fully equiaxed fine grains were observed in the weld zone. The alloy 800H is a single-phase austenitic alloy and contains a relatively small volume fraction of precipitates as the second phase [2]. The severe plastic deformation during friction welding imparts a high number of lattice defects in the form of a dislocation network in the weld zone. The presence of the strain energy and higher temperatures have resulted in the dynamic recrystallization of the deformed grains, and fine equiaxed grains are produced in the weld zone [19,20]. The average grain sizes of the base metal and weld metal are 46 ± 7 µm and 20 ± 2 µm, respectively. Higher temperatures experienced by the HAZ adjacent to the weld zone have resulted in grain growth, as can be observed from the micrographs. The HAZ has displayed higher grain size values than the base metal and weld metal, viz. 65 ± 3 µm. As indicated earlier, the alloy 800H is a solid solution strengthened FCC austenitic alloy with a small number of precipitates. In this study, the alloy 800H used to produce the welded joints was in mill annealed condition. The precipitation of Cr_23_C_6_ precipitates along the grain boundaries would require further aging treatment after the mill annealing. It can be suggested that recrystallization and grain growth need less activation energy when the microstructure contains a single phase, resulting in grain growth in the HAZ regions of the weld joints. Typical interfacial temperatures during the linear friction welding were measured using thermal imaging techniques and found to reach around 1100 °C (~0.8T_m_) for Ni-based alloys [21]. Based on the thermodynamic analysis of carbide precipitation in steels, temperatures above 1050 °C promote austenite grain growth due to the dissolution of Ti(C, N) precipitates [22]. Therefore, it can be suggested that a lack of Cr_23_C_6_ precipitates and the dissolution of Ti(C, N) precipitates in the HAZ during the welding resulted in the grain growth of the single-phase austenite as the grain boundary migration proceeded unobstructed.

The optical micrographs of the PWHT joints are compared with the as-welded micrographs in Figure 4b. It was observed that the weld zone experienced a considerable increase in grain size after the PWHT. The average grain size value of the weld zone after the PWHT was 35 ± 4 µm. A small but relative increase in the grain size in the HAZ and base metal was observed after the PWHT of the friction welded joints.

### 3.2. Mechanical Properties

**Figure 5:**
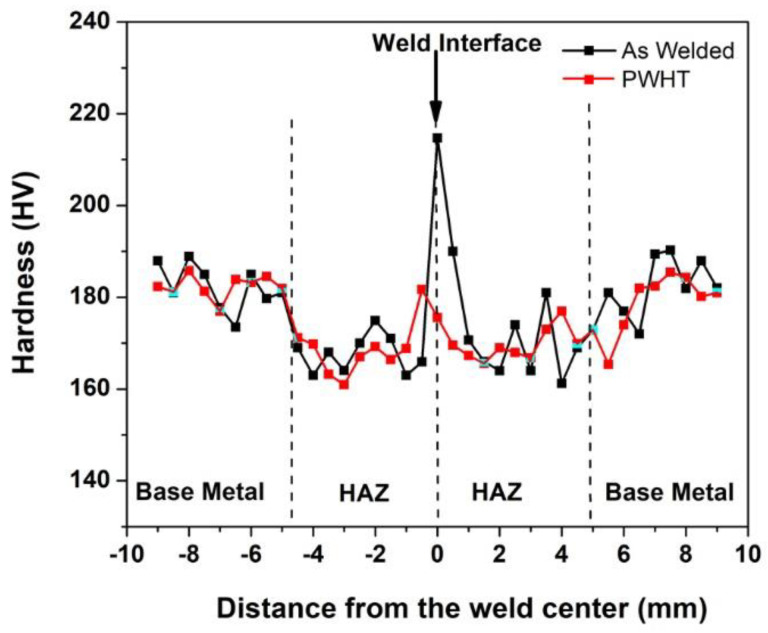
The hardness distribution across the weld interface of the alloy 800H friction weld joints in as-welded and PWHT condition.

The hardness profiles obtained across the weld interface of the as-welded and PWHT samples are shown in Figure 5.

**Figure 6:**
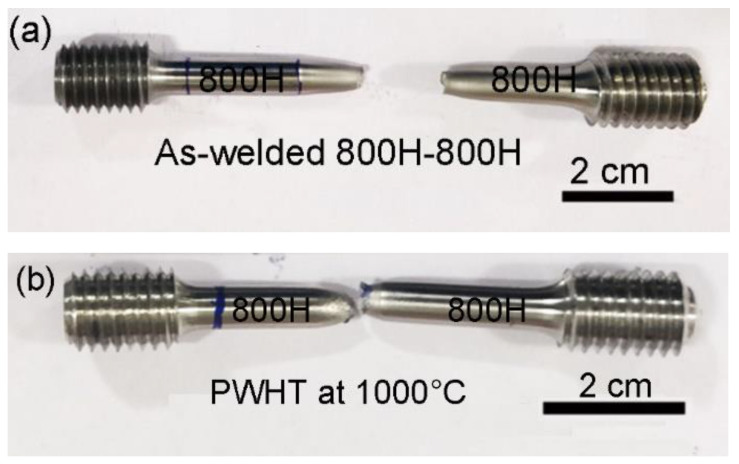
Tensile failure location of the (**a**) as-welded and (**b**) PWHT weld joints.

**Figure 7:**
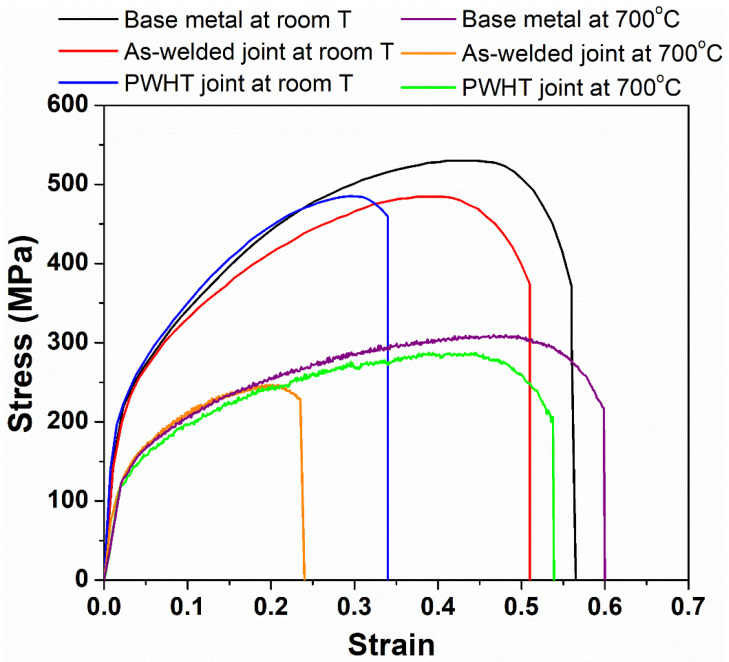
Typical tensile curves of the base metal, as-welded, and PWHT friction welded joints at room temperature and at 700 °C.

The weld zone hardness is highest in the as-welded condition compared to the HAZ and base metal conditions due to the dynamic recrystallization that occurred during the welding and to the concomitant fine grain structure [23]. The as-welded hardness value of the weld zone, 214 HV, was reduced to a lower hardness value of 181 HV as a result of PWHT. PWHT has resulted in grain growth in the weld zone and caused a decrease in hardness. However, it is worth noting that the weld zone hardness in the post-weld solution treated condition is still higher than in the HAZ. The HAZ hardness in both samples is lowest due to the higher grain size values compared to the weld metal and base metal, a result of the grain growth triggered by the peak temperatures experienced during the welding cycle [11]. The as-welded and PWHT samples failed in the HAZ region of the friction weld joint during the room temperature tensile testing. The failure location is the region of the lowest hardness across the weld joints, viz. the HAZ region with a coarse grain structure. The failure in the HAZ region indicates that the welds produced are sound and strong. Images of the samples after the tensile failure are shown in Figure 6. It can be observed that the failure location is not in the middle (weld zone) but rather slightly offset from the middle. The typical tensile curves of the base metal, as-welded, and PWHT weld joints at room temperature and at 700 °C are shown in Figure 7.

The yield strength (YS), ultimate tensile strength (UTS), and percentage of elongation (% El) of the base metal and friction weld joints in as-welded and PWHT conditions are compared in Table 3.

**Figure 8:**
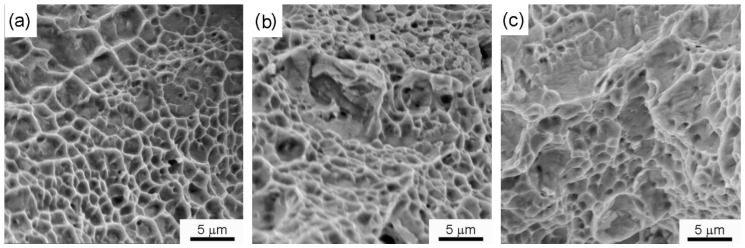
Room temperature tensile fracture morphology of (**a**) base metal, (**b**) as-welded, and (**c**) PWHT weld joints.

It can be observed from Table 3 that the weld joints in the as-welded condition displayed percentage elongation (% El) values comparable to the base metal at room temperature. A small decrease in the yield strength (YS) and ultimate tensile strength (UTS) of the as-welded and PWHT friction weld joints was observed. In contrast, a notable drop in the % El (34 ± 4%) of the PWHT friction weld joints was observed when compared to the base metal % El (56 ± 2%) and the as-welded friction weld samples (51 ± 2%). The thermal cycles during the welding and subsequent PWHT have resulted in the grain coarsening of the HAZ region, lower uniform elongation, and early necking during the room temperature tensile testing. The secondary electron micrographs of the tensile fractured surfaces of the base metal, as-welded, and PWHT weld joints are shown in Figure 8a–c, respectively. The fracture surfaces displayed fibrous features with dimples indicative of the ductile fracture. However, the post-weld solution heat-treated sample showed coarser dimples, which could be due to the coarse grain size of the failure location, viz. the HAZ region.

Lower YS and UTS values were observed at a higher temperature compared to at room temperature. The as-welded sample exhibited the lowest UTS (250 ± 2 MPa) and % El (23 ± 7%) values compared to the other high-temperature tested samples. As noted earlier, the as-welded samples displayed the lowest grain size values in the weld metal compared to the base metal and the PWHT weld joints. Grain boundary diffusion and sliding mechanisms play a prominent role at higher working temperatures during the deformation compared to at room temperature [24]. With a fine-grained weld metal, the as-welded sample exhibited lower uniform elongation and early necking during the high-temperature tensile testing because of the higher grain boundary area in the weld metal region. The localization of the deformation to a narrow fine-grained segment, viz. the weld metal, would result in early necking and a lower percentage of elongation during the testing. The localization of the deformation during the high-temperature tensile testing would also result in lower strain hardening and a lower UTS value (250 ± 2).

**Figure 9:**
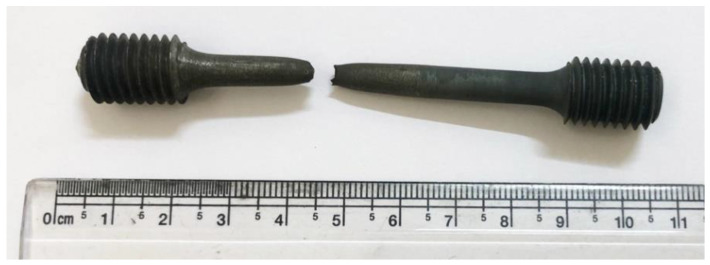
The tensile failure location of PWHT joint tested at 700 °C.

In contrast, the PWHT weld joints showed superior performance with respect to the UTS and % El values, at par with the base metal at higher temperatures compared to the as-welded weld joints. Grain coarsening achieved during the PWHT improved the high-temperature strength of the weld metal and shifted the failure location to the base metal, as shown in Figure 9. In summary, in the as-welded condition, the weld metal displayed a fine grain structure due to the dynamic recrystallization that occurred during the welding and resulted in superior room temperature mechanical properties. However, the fine-grained structure of the weld metal caused an early failure with lower strength at room temperature. The weld metal grain coarsening due to the PWHT has resulted in superior high-temperature properties compared to the as-welded joints.

## 4. Conclusions

The rotary friction welds of the alloy 800H cylindrical rods were successfully fabricated, and a PWHT was carried out on the weld joints. Microstructural and mechanical properties investigations were conducted for the as-welded and post-weld solution-treated weld joints. The following conclusions were drawn:

1. The lowest grain size value was observed in the weld zone of the as-welded friction weld joints due to the dynamic recrystallization of the heavily strained grains in the plasticized region of the joint.
2. The HAZ region experienced substantial grain growth due to the higher temperatures and the lack of precipitates in the single-phase austenite matrix.
3. Post-weld solution heat treatment resulted in grain growth with a decrease in the peak hardness in the weld zone. The average grain size weld metal in the as-welded condition was 20 ± 2 μm. In contrast, the weld metal grain size of the PWHT joints showed a higher grain size value of 35 ± 4 µm.
4. Tensile specimens failed in the HAZ region for both as-welded and post-weld solution-treated weld joints due to the lower hardness value of the HAZ region as compared to the weld zone and base metal.
5. The PWHT weld joints showed the improved high-temperature strength of the weld metal and shifted the failure location to the base metal.
6. The PWHT resulted in a higher UTS and in percentage elongation values at higher temperatures compared to the as-welded joints. At high temperatures, grain boundaries are the weakest zones and play a major role in the deformation of the material. The weld metal with a higher grain size and a consequently lower grain boundary area displayed better mechanical performance at high temperatures.

## Figures and Tables

**Table 1:** The chemical composition (wt.%) of the base metal alloy 800H.

Elements	Ni	Cr	C	Mn	Si	Al	Ti	Fe
Alloy 800H	31.3	21.1	0.08	0.61	0.28	0.3	0.3	bal.

**Table 2:** Friction welding parameters.

Parameters	Explored Parameter Range	Final Chosen Parameter
Friction pressure (MPa)	50–200	200
Burn-off length (mm)	2–6	4
Upset pressure (MPa)	100–400	400
Upset time (s)	4 (held constant)	4
Spindle speed (rev/min)	2000 (held constant)	2000

**Table 3:** The results of room-temperature and high temperature (700 °C) tensile testing of the alloy 800H base metal, as-welded, and PWHT weld joints (average properties from three tests in each case).

Sample	Yield Strength (MPa)	Ultimate Tensile Strength (Mpa)	Elongation %	Failure Location
Base Metal-RT *	201 ± 5	529 ± 6	56 ± 2	-
As-welded-RT	198 ± 5	484 ± 6	51 ± 2	HAZ
PWHT-RT	185 ± 8	485 ± 7	34 ± 4	HAZ
Base Metal-HT ^#^	110 ± 4	319 ± 3	60 ± 5	-
As-welded-HT	112 ± 2	250 ± 2	23 ± 7	WM
PWHT-HT	130 ± 1	300 ± 6	54 ± 2	BM

* RT: Room Temperature, ^#^ HT: High temperature 700 °C.

## Data Availability

Not applicable.
